# Cholesterol Gallstone among Patients with Cholelithiasis Admitted to the Department of Surgery of a Tertiary Care Centre

**DOI:** 10.31729/jnma.8362

**Published:** 2023-12-31

**Authors:** Ashwini Kumar Jha, Mina Jha, Sunil Adhikari

**Affiliations:** 1 Department of Surgery, Janaki Medical College and Teaching Hospital, Janakpur Dham, Dhanusha, Nepal; 2 Department of Anatomy, Janaki Medical College and Teaching Hospital, Janakpur Dham, Dhanusha, Nepal; 3 Department of Radiology, Janaki Medical College and Teaching Hospital, Janakpur Dham, Dhanusha, Nepal

**Keywords:** *cholesterol*, *gallbladder*, *gallstone*, *prevalence*

## Abstract

**Introduction:**

Gallstone is one of the most common pathological conditions found mostly in females. The incidence of cholesterol gallstones is developing higher nowadays which is increasing the burden of cost in society. This study aimed to find out the prevalence of cholesterol gallstones among patients with cholelithiasis admitted to the Department of Surgery of a tertiary care centre.

**Methods:**

A descriptive cross-sectional study was conducted among patients with cholelithiasis who presented in the Department of Surgery of tertiary care centre for cholecystectomy (laparoscopic or open) from 23 December 2022 to 22 September 2023 after obtaining ethical approval from the Institutional Review Committee. Patients who were diagnosed by use of abdominal ultrasound or CT scan were included. Those patients with gallbladder polyps, cholangitis, and gallbladder tumours were excluded from the study. A convenience sampling method was used. The point estimate was calculated at a 95% Confidence Interval.

**Results:**

Among 190 patients, the prevalence of cholesterol gallstones was seen in 85 (44.74%) (37.6751.81, 95% Confidence Interval). The female to male ratio was 6.72:1.

**Conclusions:**

The prevalence of cholesterol gallstones was found to be higher as compared to other studies done in similar settings.

## INTRODUCTION

One of the most common biliary pathologies is gallstone.^1^ Its worldwide prevalence is 3-21.9%, in Asia 4-15% and overall prevalence in Nepal is 4.87%.^2,3^ Gallstones can be divided into three types: cholesterol, pigment (brown/black) or mixed stones.^1^

Cholesterol gallstones are found in more than 80% of patients with cholelithiasis, and the pathogenesis includes both local (that is, gallbladder and bile) and systemic factors.^4^ Family history, fertility, climate and dietary conditions may also be related to increased incidence of cholesterol gallstone formation in Asia.^5^ In Nepal, the incidence of gallstones is increasing causing one of the major operative conditions. Yet there are only a few studies to support it.

This study aimed to find out the prevalence of cholesterol gallstones among patients with cholelithiasis admitted to the Department of Surgery of a tertiary care centre.

## METHODS

A descriptive cross-sectional study was conducted among patients admitted with cholelithiasis presenting to the Department of Surgery, Janaki Medical College and Teaching Hospital, Janakpur Dham, Dhanusha, Nepal from 23 December 2022 to 22 September 2023 after obtaining Ethical approval from the Institutional Review Committee of the same institute (Reference number: IRC/24/2079-80). They were assured of confidentiality and informed written consent was taken. All patients with gallstones who presented to the Department of Surgery either from the emergency or outpatient department giving informed consent were included in the study. Patients with gallbladder polyp, Cholangitis, choledocholithiasis, gallbladder tumour and other gallbladder diseases were excluded. A convenience sampling method was adopted. The sample size was calculated by using the following formula:


$$\begin{array}{ll} \text{n} & ={\text{Z}}^{2}\times \frac{\text{p}\times \text{q}}{{\text{e}}^{\text{2}}}\\ & =1.96^{2}\times \frac{0.50\times 0.50}{0.08^{2}}\\ & =151 \end{array}$$

Where,

n = minimum required sample size
Z = 1.96 at 95% Confidence Interval (CI)
p = prevalence taken as 50% for maximum sample size calculation
q = 1-p
e = margin of error, 8%

The minimum sample size calculated was 151. However, 190 patients were included in the study.

The presence of gallstones was determined by ultrasonography or computed tomography (CT) scan. After the cholecystectomy operation (laparoscopic or open), removed gallstones were identified by the general surgeon as cholesterol gallstones based on external appearance.^6^

Data were entered and analyzed using Microsoft Excel 2016. The point estimate was calculated at a 95% CI.

## RESULTS

Among 190 patients, the prevalence of cholesterol gallstones was seen in 85 (44.74%) (37.67-51.81, 95% CI) patients. Among patients with cholesterol gallstones, 11 (12.94%) were male and 74 (87.06%) were females. A total of 62 (72.94%) were Hindu, and 23 (27.06%) were Muslim (Table 1).

**Table 1:** Sociodemographic characteristics of the patients with cholesterol gallstones (n= 85).

Characteristics	Category	n (%)
Sex	Male	11 (12.94)
	Female	74 (87.06)
Marital status	Married	80 (94.12)
	Unmarried	5 (5.88)
Religion	Hindu	62 (72.94)
	Muslim	23 (27.06)

The prevalence of cholesterol gallstones was common in the age group of 16-30 years seen in 41 (48.23%), followed by 31-40 years seen in 26 (30.59%) (Table 2).

**Table 2:** Age distribution among patients with cholesterol gallstones (n= 85).

Age (years)	n (%)
16-30	41 (48.23)
31-40 years	26 (30.59)
41-50 years	13 (15.29)
51-60 years	4 (4.71)
61-70 years	1 (1.18)

Out of the total patients, 21 (24.71%) were vegetarian and 64 (75.29%) were consuming a mixed type of diet. Among patients with cholesterol gallstones, 13 (15.29%) were smokers. A total of 79 (92.94%) had multiple stones revealed by abdominal ultrasonography (USG) (Table 3).

**Table 3:** Abdominal USG findings among patients with cholesterol gallstones (n= 85).

USG finding	n (%)
Single stone	5 (5.88)
Multiple stone	79 (92.94)
Mucocele	1 (1.18)

A total of 84 (98.82%) patients underwent laparoscopic cholecystectomy and only 1 (1.18%) patient underwent conversion from laparoscopic cholecystectomy to open cholecystectomy.

## DISCUSSION

Gallstones are a major public health problem in all developed countries. In the present study, the prevalence of cholesterol gallstones was 85 (44.74%) among the patients with cholelithiasis admitted to the surgery department of the tertiary care centre. A similar result constituting 40.4% of cholesterol stone had been reported in a study conducted in Baghdad.^7^ In contrast to the study conducted in a medical college in Jharkhand, India, the prevalence of cholesterol gallstones was 98%,^8^ which is higher than the present study. Another study was conducted in Uttar Pradesh, India^9,10^ and in the central part of Nepal^12^ revealing 15.3%, 16% and 12.5% of cholesterol gallstones respectively. These studies showed less prevalence of cholesterol gallstones than the present study.

Cholesterol gallstones are closely related to gallbladder dysfunction which causes bile to stay longer in the gallbladder, resulting in nucleation and crystallization and finally leading to stone formation.^11^

In our study, male to female ratio was 1:6.7 which was similar to the study done in Uttar Pradesh, India.^9^ The mean age of the patient was 32.63±9.35 years which was comparable with the study done in a medical college of Nepal.^12^ Various kinds of the literature showed maximum prevalence in the age group 31-40,^5,12^ however, the present study showed the maximum incidence of cholesterol gallstones was 48.23% in the 16-30 years age group. This difference may be due to early marriage in young girls of the rural area leading to pregnancy at an early age in this age group and incidental finding of gallstones on ultrasound during pregnancy. Also, the dietary fats and environment affect the formation of gallstones. In this study, 21 (24.71%) consumed vegetarian diet while 64 (75.29%) consumed non-vegetarian diet.

One of the useful diagnostic tools used to diagnose gallstones is abdominal ultrasonography. In the present study multiple stones were seen in 79 (92.94%), single stones seen in 5 (5.88%) cholesterol gallstone and 1 (1.18%) showed mucocele gallbladder. A study conducted in the northern area of Melbourne revealed that those having multiple stones were more likely to progress into gallbladder cancer.^13^ Another study in Greece concluded that one of the major predictors for severe inflammation was single gallstones and younger age.^14^

Laparoscopic cholecystectomy (LC) is a minimally invasive and commonly performed surgical procedure in developed countries.^15^ For the management of symptomatic cholelithiasis, laparoscopic cholecystectomy is considered as "the gold standard".^16^ Nowadays, in a developing country like Nepal laparoscopic cholecystectomy is becoming the treatment of choice. The conversion rate of laparotomy for uninflamed gallbladder disease ranges from 2% to 15%, and in acute calculus cholecystitis cases, from 6% to 35%.^17^ In our study only 1 (1.18%) case got conversion from laparoscopic to open cholecystectomy due to a severely contracted gallbladder and frozen Calot's triangle.

This was a single-centered study with a limited time frame so it cannot be generalized to the larger population.

## CONCLUSIONS

The prevalence of cholesterol gallstones is higher than in other studies done in similar settings.
